# A Psychometric Tool for a Virtual Reality Rehabilitation Approach for Dyslexia

**DOI:** 10.1155/2017/7048676

**Published:** 2017-02-13

**Authors:** Elisa Pedroli, Patrizia Padula, Andrea Guala, Maria Teresa Meardi, Giuseppe Riva, Giovanni Albani

**Affiliations:** ^1^Applied Technology for Neuro-Psychology Lab, Istituto Auxologico Italiano, Via Magnasco 2, 20149 Milano, Italy; ^2^Department of Paediatrics, Ospedale Castelli, Via Crocetta, 28921 Pallanza, Italy; ^3^Department of Child Neuropsychiatry, Ospedale Castelli, Via Crocetta, 28921 Pallanza, Italy; ^4^Department of Psychology, Catholic University of Milan, Milan, Italy; ^5^Division of Neurology and Neurorehabilitation, IRCCS Istituto Auxologico Italiano, Via Cadorna 90, Piancavallo, Verbania, Italy

## Abstract

Dyslexia is a chronic problem that affects the life of subjects and often influences their life choices. The standard rehabilitation methods all use a classic paper and pencil training format but these exercises are boring and demanding for children who may have difficulty in completing the treatments. It is important to develop a new rehabilitation program that would help children in a funny and engaging way. A Wii-based game was developed to demonstrate that a short treatment with an action video game, rather than phonological or orthographic training, may improve the reading abilities in dyslexic children. According to the results, an approach using cues in the context of a virtual environment may represent a promising tool to improve attentional skills. On the other hand, our results do not demonstrate an immediate effect on reading performance, suggesting that a more prolonged protocol may be a future direction.

## 1. Introduction 

Dyslexia affects the ability to obtain accurate and fluent reading skills in children without neurological impairment who have a normal IQ score. Among Italian young people, the reported prevalence of dyslexia is between 2.5% and 3.5% [1]. A student with some reading problems in the 1st grade has a 90% probability of showing the same deficit in the 4th grade and 75% in high school [2]. In dyslexia, both genetics and environmental factors are important and strongly related. Twin studies have shown low heritability in countries with low levels of reading education, while high heritability has been found in those countries with higher levels [3]. However, the importance of genetic factors is about 54% to 75%, with 68% for identical twins and 50% for children having relatives with dyslexia [2]. A combination of many genetic and environmental factors probably causes dyslexia [4].

Subjects with dyslexia may show specific patterns of atypical brain activation during reading or phonological tasks. For example, one patient presents hypoactivation of the left temporoparietal cortex, the left prefrontal cortex, and the left fusiform gyrus. Structural MRI studies show several abnormalities such as a decrease in grey matter volume, reduced cerebral white matter gyrification, or a bigger corpus callosum [5, 6].

Children with dyslexia may exhibit deficits in both accuracy and speed during reading tasks. Usually, if a child reads slowly, he can make fewer mistakes. Besides, children with dyslexia may show poor phonological skills. Initially, this deficit may involve the pronunciation of words. Different expressions of phonological difficulty depend on different kinds of orthographies (consistent or less consistent orthographies) [2, 7].

Dyslexia is a chronic problem that affects the life of subjects and often influences their life choices. Only a few dyslexic patients decide to continue studies in higher education and those who do may face several difficulties during the years. From the psychological point of view, some students do not want to be recognized as dyslexic in order to avoid discrimination from both professors and other students. This attitude leads the student to reject the use of the modifications provided by the school. Other learners may refuse the modifications to prove that they are able to complete the tests as easily as all the other students. Generally, dyslexic students prefer professors who use innovative formats and provide access to the study material before the lessons and in several formats. Obviously, these students prefer oral assessment because it allows them to present their best [8]. Technology offers great tools to support dyslexic children in their educational careers; Universal Design for Learning (UDL) can represent the future of schools [9], because it helps dyslexic students to achieve their educational goals.

Many dyslexic adults may have difficulty in finding an adequate job. As de Beer et al. [10] showed in their review, the structured corporate environment is stressful and not supportive of dyslexic employees. In this kind of work environment, the positive characteristics of dyslexic people (creativity, solving problems, and persistence) may be overshadowed by the negative ones (problems in reading or writing, slowness, and negative feelings about dyslexia). It often happens that dyslexic people become entrepreneurs in order to open a new business and work for themselves.

Unfortunately, there is no agreement about the gold standard in the rehabilitation of dyslexia. Regardless of the method, it is clear that the best treatment includes intensive sessions, explicit instructions for the exercises, single person or small group implementation, and starting as soon as possible, even before the diagnosis if possible. It is also important to give special attention to the phonological awareness and compensative strategies [11].

All the standard methods use a classic paper and pencil format but the exercises are boring and demanding for children who may have difficulty in completing the treatments [12]. It is crucial to develop a new rehabilitation program that could help children with their problem in a funny and engaging way. Franceschini and colleagues used a Wii-based game in order to demonstrate that a short treatment with an action video game, rather than phonological or orthographic training, may improve the reading abilities in dyslexic children. They showed that training attentional skills improved the reading speed without reducing the accuracy in reading [12].

Several current rumors about Virtual Reality mean an explosive mix of several disciplines for those who worked in the field for several years [13]. There is a natural trade-off between technological complexity and psychological needs, particularly when speaking about children. With this study, we aimed to plan a platform for an experimental task, complete enough to catch a behavioral parameter and useful for a psychological analysis of dyslexic children.

In building such a platform, we opted for a contactless solution, that is, with the sensor posed in front of the participant instead of an accelerometer or other sensors worn by the participants. This allowed us to keep the experimental setting as unobtrusive and transparent as possible for the children. The technical solution we adopted was the use of a Kinect, possible thanks to the use of NeuroVirtual 3D software, through the VRPN interface [14]. The approach and software already had been used in a clinical setting, for the Neglect [15], and explained both computational properties [16] and behavioral aspects [17–20].

The use of NeuroVirtual 3D allowed us to program automatic gesture recognition, involving the children in an active experimental setting and eliciting the needed psychological engagement for this kind of population.

Moreover, the automatic classification, recognition, and recording of participants' behavioral responses are in line with the computational psychometric paradigm highlighted by Cipresso in his seminal paper [21], where he used a Virtual Classroom to verify the idea.

Adams and colleagues proposed a Virtual Classroom as a tool to increase motivation in children with attentional deficit [22], while some authors proposed VR as a test to identify the visuospatial strengths in dyslexia [23]. In the present study, we propose a Virtual Reality training for attention in order to improve the reading skills in a sample of dyslexic children.

## 2. Methods

### 2.1. Sample

Ten children (2 females and 8 males) were analyzed at the Ospedale Castelli of Verbania.

The criteria for participation were the following: (1) age between 9 and 12 years; (2) diagnosis of dyslexia (with/without dysgraphia and dyscalculia). The exclusion criteria were the following: (1) comorbidity with other neuropsychiatric disorders (ADHD, etc.); (2) motor impairment that would not allow the interaction with the system; (3) other neurological or psychiatric diseases.

These subjects were 10.6 + 1.4 years old (mean + standard deviation) and had 5.6 + 1.3 years of education. The demographic characteristics of each participant are reported in Table 1.

At the time, there was no control group.

### 2.2. Materials

#### 2.2.1. Hardware

The VR system consisted of a standard laptop and a motion sensing input device, Microsoft's Kinect, and an audio system. The tracking system consisted of an infrared tracking system camera, which was placed on the table near the laptop.

#### 2.2.2. Software

**Figure 1:**
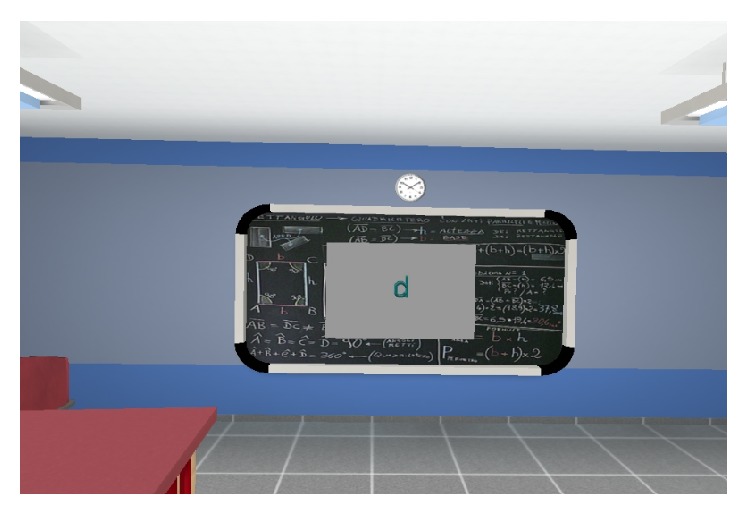
Example of the environment of task 2.

All the virtual environments were developed with NeuroVR. This new software allows normal users to customize several virtual environments according to their needs. Users did not need to have programming skills; all the objects easily were placed into the preexisting environments using an icon-based interface. The tasks took place in the same Virtual Classroom, where patients were sitting at a desk and looking at the blackboard (Figure 1).

All the visual stimuli were shown on the blackboard and the tasks were explained by a voice. All the instructions were presented before the tasks and repeated until the child understood.

To respond to a target, the children had to raise one extended arm laterally and stop when the hand reached shoulder height.

All subjects had to do three different tasks.

In the first one, they had to respond when a target appeared on the blackboard within a series of objects. All the stimuli were 3D objects and the target changed in every session.

During the second task, different letters were presented on the blackboard and the children had to respond only when one letter (i.e., the letter “G”) appeared after another letter (i.e., the letter “A”).

In the last exercise, the patients listened to a story and had to respond only when one of the four colors on the blackboard had been named related to a given category (i.e., “clothes”).

### 2.3. Procedure

**Figure 2:**
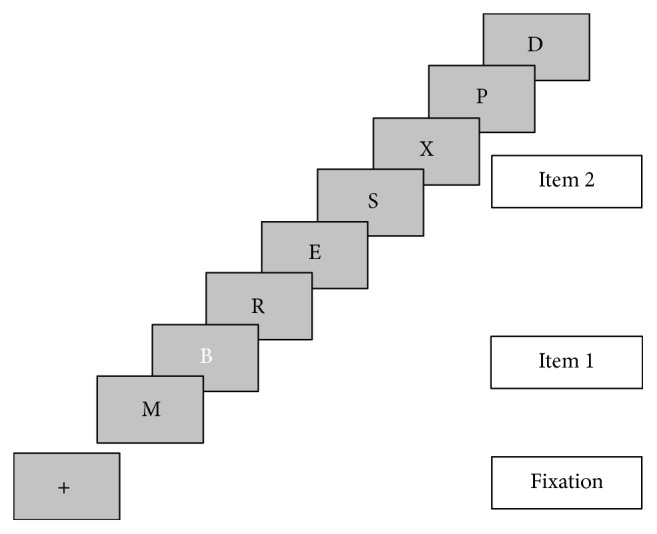
Experimental paradigm of ABT.

**Figure 3:**
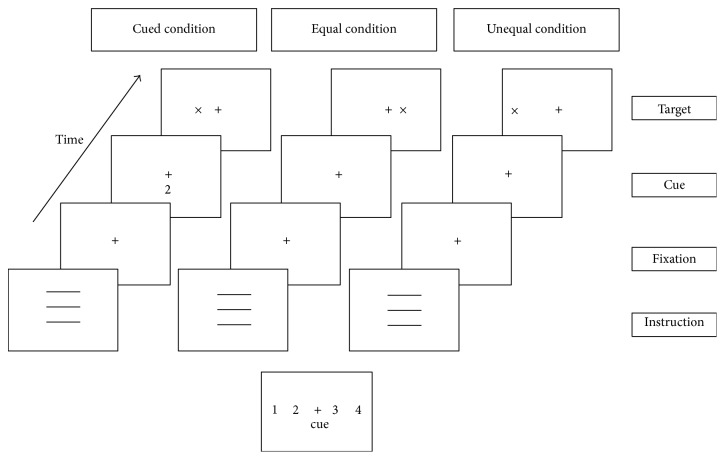
Experimental paradigm of Posner's task.

In order to assess the reading skills and the level of attention before and after the training, three tasks were used:

- Reading test of words and not words: patients had to read several lists of words with different characteristics: words or not words, short or long, and high or low frequency.
- Attentional Blink Task (ABT- [24]): patients had to see a series of letters and then answer two questions: “Which letter is white: B, L, S?” “There is the letter “X”?” (Figure 2)
- Posner's task [25]: participants had to press a button when a target (X) appeared on the screen. This could happen in three different situations. In the first type of task, the location of the target was identified by a number (that indicated the location of the target) before each single target presentation under the fixation point. In the second type of task, the probable position of the target was shown before the presentation of all targets. In the last one, there was no cue about the position of the target (Figure 3).

In order to assess the usability, the “System Usability Scale” (SUS, [26]) was submitted at the end of the four weeks of treatment.

The group received the training for 30–45 minutes a day, 2 days a week, for four weeks.

All the tasks took place in a virtual environment that reproduced a classroom; the child was sitting at a bench and could interface with the blackboard to carry out certain tasks that the teacher's voice explained.

All the sessions took place in a quiet room in the hospital and the environment was controlled to try to limit all potential distractors. Participants were seated 2 m in front of a laptop and Kinect.

### 2.4. Data Analysis

Data were entered into Microsoft Excel and analyzed using SPSS for Windows, version 18.0 (Statistical Package for the Social Sciences (SPSS) for Windows, Chicago, IL, USA). Because of the small sample size, the Wilcoxon signed rank sum test was used to compare scores in the above described measures before and after our virtual training. There was no missing data. The level of significance was set at *α* = 0.05.

## 3. Results

Table 2 provides a comprehensive overview of the results obtained from the analysis of the attentional tasks.

As is possible to see in Table 2, all attentional indexes, except one, have been significantly improved after the administration of the virtual training. There is a significant decrease in two of the three indexes in Posner's tasks: “cued target” (*Z* = −2.090; *p* < 0.037) and “unequal target” (*Z* = −1.988; *p* < 0.047). These results suggest that there is an improvement only in the conditions in which a suggestion is provided. Also, there is a trend to significance in the accuracy of the Attentional Blink Test (*Z* = −1.770, *p* < 0.077).

Concerning the reading tasks, Table 3 offers a detailed synthesis of the results.

As is possible to see in Table 3, most of the indexes from the reading tasks have not been significantly improved after the administration of our virtual training. Interestingly, an almost significant decrease was found in the time of reading low frequency long words (*Z* = −1.684; *p* < 0.092).

All the subjects finished the VR session tasks.

After four weeks of treatment, all the patients showed a significant improvement in the attentional domain, as shown in Table 1.

Specifically, patients improved significantly in the mean time for two conditions of Posner's task: “cued target condition” and “unequal target condition.”

No significant difference emerged in analyzing the reading test before and after treatment.

The “low frequency long word” list showed a “tendency to significance” that indicated a slight improvement.

## 4. Discussion 

To our knowledge, the present study represents the first use of VR for rehabilitation of reading deficits in dyslexia, by potentiating attention abilities. According to our results, the use of cues in the context of a virtual environment may represent a promising approach to improve attentional skills. Attention is a crucial target in the rehabilitative treatment of these patients and may represent a first step toward a phonological awareness and an improvement of decoding skills. However, all the most cited theories about the origin of dyslexia converge about a functional deficit in the afferent pathways required for the verbal output.

The “rapid temporal processing theory” hypothesizes a deficit in auditory temporal processing that compromises the discrimination of language sounds. This deficit is stronger when there are very brief differences in the auditory inputs. Seven percent of preschool children may show a deficit in phonological awareness and/or morphosyntax and may have difficulty in identifying the order of rapidly presented tones. Students who exhibit these problems are those most likely to develop dyslexia [27, 28]. The “magnocellular hypothesis” is based on the observation that dyslexic people may have a problem in distinguishing the letters and their order. According to this theory, this problem is caused by an abnormality in visual magnocellular nerve cells. This pathway is important to control the visual guidance of attention and eye fixations. Dyslexic children may have problems with processing rapidly changing visual nonverbal information. The severity of this deficit is an indicator of the level of future reading deficits [2, 29].

While our results do not demonstrate an immediate effect on reading performance, suggesting that a more prolonged protocol may be a future direction, the presence of a significant decrease in the time of reading low frequency long words supports the possible validity of the proposed approach.

Indeed, according to Torgesen [30], rehabilitation training must focus on phonological awareness and decoding tasks; the program has to be intensive (almost an hour per day for 2 months) and must involve few students. Dyslexic and poor reader children may show phonological difficulties as early as kindergarten. Through an individual screening, based on the knowledge of letter names and sounds, phonological awareness, and speed of naming, it is possible to predict the future reading ability. Thus, it is important that an intensive phonological rehabilitation program be proposed for the high-risk students. This kind of exercise is challenging and could be helpful for many children but not for all.

Our protocol is innovative because it uses Virtual Reality to improve attentional skills in children with dyslexia. This technology is able to involve children in nonconventional tasks and allow them to work in a virtual environment similar to the real one.

Other studies are needed in order to clarify the effect of this protocol on the deficits associated with dyslexia, but the results of our pilot study and the achieved effect above 0.6 (effect size* dz* calculated with GPower software) are promising, even if the small sample size highlights the need of further studies.

Finally, we would like to stress the computational aspects of our study. First of all, the protocol and platform used are based on computational aspects of neuropsychological tests, since the platform was automatized and structured with automatic gesture recognition. On the other hand, the recording of the gestures and the participants' behavior open the way to Cipresso's paradigm of computational psychometrics to model behavior through the use of Virtual Reality [21]. Here, due to the small sample size and the different purpose, we did not consider the dynamics of the behavior, even if in the future this could represent a natural evolution of the protocol. Other computational aspects to be considered at the moment in a perspective view are to use sophisticated algorithms of machine learning for the best big behavioral data extraction and information toward an improved platform for helping the target population based on structured and solid data.

## Figures and Tables

**Table 1:** Demographic characteristics of the sample.

ID	Gender	Age (years)	Education (years)
1	M	12	7
2	M	9	4
3	F	12	7
4	M	9	4
5	M	9	4
6	M	11	6
7	M	10	6
8	M	12	6
9	M	12	7
10	F	10	5

**Table 2:** Results of attentional tests.

	Index	T1	T2	*Z*	*p*
		Mean (SD)	Mean (SD)		
Posner's test (milliseconds)	Cued target	379.72 (90.88)	435.78 (83.28)	−2.090	0.037
	Mean time				
	Equal target	355.78 (50.48)	389.61 (69.66)	−1.274	0.203
	Mean time				
	Unequal target	370.48 (66.23)	416.94 (74.82)	−1.988	0.047
	Mean time				

Attentional Blink Test	Accuracy	90.95 (15.94)	87.62 (13.13)	−1.770	0.077

**Table 3:** Results of “reading test of words and not words.”

	Index	T1	T2	*Z*	*p*
		Mean (SD)	Mean (SD)		
Short nonword	Errors	5.70 (4.11)	5.30 (3.06)	−.526	0.599
	Time (seconds)	42.20 (9.31)	39.40 (10.61)	−1.228	0.219
Long nonword	Errors	8.00 (3.27)	7.40 (2.32)	−.307	0.759
	Time (seconds)	70.50 (11.32)	65.30 (15.49)	−1.612	0.107
High frequency short word	Errors	1.20 (1.4)	1.30 (1.42)	−.378	0.705
	Time (seconds)	27.40 (8.61)	27.60 (9.09)	−.356	0.722
High frequency long word	Errors	2.60 (1.84)	2.40 (1.9)	−.520	0.603
	Time (seconds)	40.90 (15.99)	38.40 (13.962)	−1.277	0.202
Low frequency short word	Errors	3.40 (2.07)	3.60 (2.07)	−.299	0.765
	Time (seconds)	37.80 (9.52)	35.30 (10.54)	−1.555	0.120
Low frequency long word	Errors	7.10 (3.7)	5.70 (2.67)	−1.427	0.154
	Time (seconds)	63.40 (20.91)	57.60 (17.87)	−1.684	0.092
